# Behavioral Time Scale Plasticity of Place Fields: Mathematical Analysis

**DOI:** 10.3389/fncom.2021.640235

**Published:** 2021-03-01

**Authors:** Ian Cone, Harel Z. Shouval

**Affiliations:** ^1^Department of Neurobiology and Anatomy, University of Texas Medical School, Houston, TX, United States; ^2^Applied Physics Program, Rice University, Houston, TX, United States

**Keywords:** synaptic plasticity, mathematical model, place field, hippocampus, eligibility trace

## Abstract

Traditional synaptic plasticity experiments and models depend on tight temporal correlations between pre- and postsynaptic activity. These tight temporal correlations, on the order of tens of milliseconds, are incompatible with significantly longer behavioral time scales, and as such might not be able to account for plasticity induced by behavior. Indeed, recent findings in hippocampus suggest that rapid, bidirectional synaptic plasticity which modifies place fields in CA1 operates at behavioral time scales. These experimental results suggest that presynaptic activity generates synaptic eligibility traces both for potentiation and depression, which last on the order of seconds. These traces can be converted to changes in synaptic efficacies by the activation of an instructive signal that depends on naturally occurring or experimentally induced plateau potentials. We have developed a simple mathematical model that is consistent with these observations. This model can be fully analyzed to find the fixed points of induced place fields and how these fixed points depend on system parameters such as the size and shape of presynaptic place fields, the animal's velocity during induction, and the parameters of the plasticity rule. We also make predictions about the convergence time to these fixed points, both for induced and pre-existing place fields.

## 1. Introduction

Experiments and models of synaptic plasticity have, for several decades, concentrated on plasticity which depends on coincident or nearly coincident activation of pre- and postsynaptic cells. This is most clearly exemplified by spike timing dependent plasticity (STDP), in which timing differences between pre- and postsynaptic spikes, on the order of tens of milliseconds, significantly impact the sign and magnitude of synaptic plasticity (Markram et al., 1997; Bi and Poo, 1998; Shouval, 2010), while co-activation of pre- and postsynaptic activity at larger intervals produces no synaptic plasticity. Such correlations between pre- and postsynaptic activity are possible biological implementations of unsupervised learning (Kempter et al., 1999; Song et al., 2000). However, many aspects of behavioral plasticity depend on a supervising signal, or a reward, which can occur with delays that range from hundreds of milliseconds to seconds or more.

Recent plasticity experiments in hippocampus *in vivo* (Bittner et al., 2015, 2017; Milstein et al., 2020) have shown place-field plasticity that occurs rapidly in response to either naturally occurring or artificially induced dendritic calcium spikes, also known as “plateau potentials.” These protocols have shown both an increase and a decrease in synaptic efficacies occurring in synapses that were active seconds before or after the plateau potentials. This plasticity, coined “behavioral timescale synaptic plasticity” (BTSP), is therefore unable to be reconciled with forms of synaptic plasticity that depend on tight correlations between pre and postsynaptic activity.

The difficulty in associating events (such as stimulus and reward) at larger time scales is called the temporal credit assignment problem (Sutton and Barto, 2018). Various methods to solve the temporal credit assignment problem have been proposed, none of which solely depend on coincidences on the range of tens of milliseconds. One possible solution depends on synaptic eligibility traces, which can last for several seconds following neural activity, and which can be converted into changes in synaptic efficacies if they are followed by a reward or an instructive signal (Izhikevich, 2007; Gerstner et al., 2018). This is similar to synaptic tagging (Frey and Morris, 1997), but the dynamics of eligibility traces are on the order of seconds rather than hours, and seconds are the time scale relevant for place field plasticity. Recent evidence in several systems has provided experimental support for the existence of synaptic eligibility traces. It has been shown that a neuromodulator applied seconds after a pre before post pairing protocol can induce long-term potentiation (LTP), and that the magnitude of plasticity depends on the delay between the stimulus and application of neuromodulator (Yagishita et al., 2014; He et al., 2015; Fisher et al., 2017; Shindou et al., 2019). It has also been shown that after a post before pre pairing protocol, a different neuromodulator can induce long-term depression (LTD) (He et al., 2015). These results suggest that pairing of pre- and postsynaptic activities can generate some currently undetermined biochemical processes, which last for several seconds, and are the substrates of the synaptic eligibility traces for LTP and LTD. If a neuromodulator is applied while the trace is sufficiently active, either LTP or LTD is induced, depending on the details of the pairing protocol. Note that in these experiments, the traces induced depend on both pre- and postsynaptic activity, while the conversion of these traces into efficacy changes depends on a third factor, a neuromodulatory signal. These examples are therefore examples of three-factor learning (Gerstner et al., 2018). Theoretical models consistent with these experimental observations have been shown to be useful in accounting for learning in model networks (Gavornik et al., 2009; He et al., 2015; Huertas et al., 2016).

The phenomenon of BTSP may also depend on synaptic eligibility traces, both for LTP and LTD. A recent paper has shown that for BTSP, these traces likely depend only on presynaptic activity and the magnitude of the existing synaptic efficacy, and that change in synaptic efficacies can depend on the overlap between these traces and an instructive signal that is activated by the plateau potential(Milstein et al., 2020). The data therefore supports a two-factor model in which the two factors are presynaptic activity and an instructive signal.

The model for BTSP we present and analyze here extends these previous results. We show that the place fields produced by a two-factor eligibility trace model have fixed points, and that these fixed points can be defined and calculated. Our model additionally predicts the convergence rate to these fixed points. In some simple cases these fixed points can be fully solved analytically. Using these solutions, we show how these fixed points depend on the system's parameters such as the shape of the presynaptic place fields and the animal's velocity. We show explicitly that the place fields become broader if the animal has a higher velocity during induction, and predict that LTD far away from the instructive signal has a slow convergence time to the fixed point. These results agree with, and extend upon, existing experiments on BTSP (Bittner et al., 2015, 2017; Milstein et al., 2020) and are achieved by a simple and analytically tractable mathematical model.

## 2. Methods and Results

### 2.1. Model Setup

**Figure 1:**
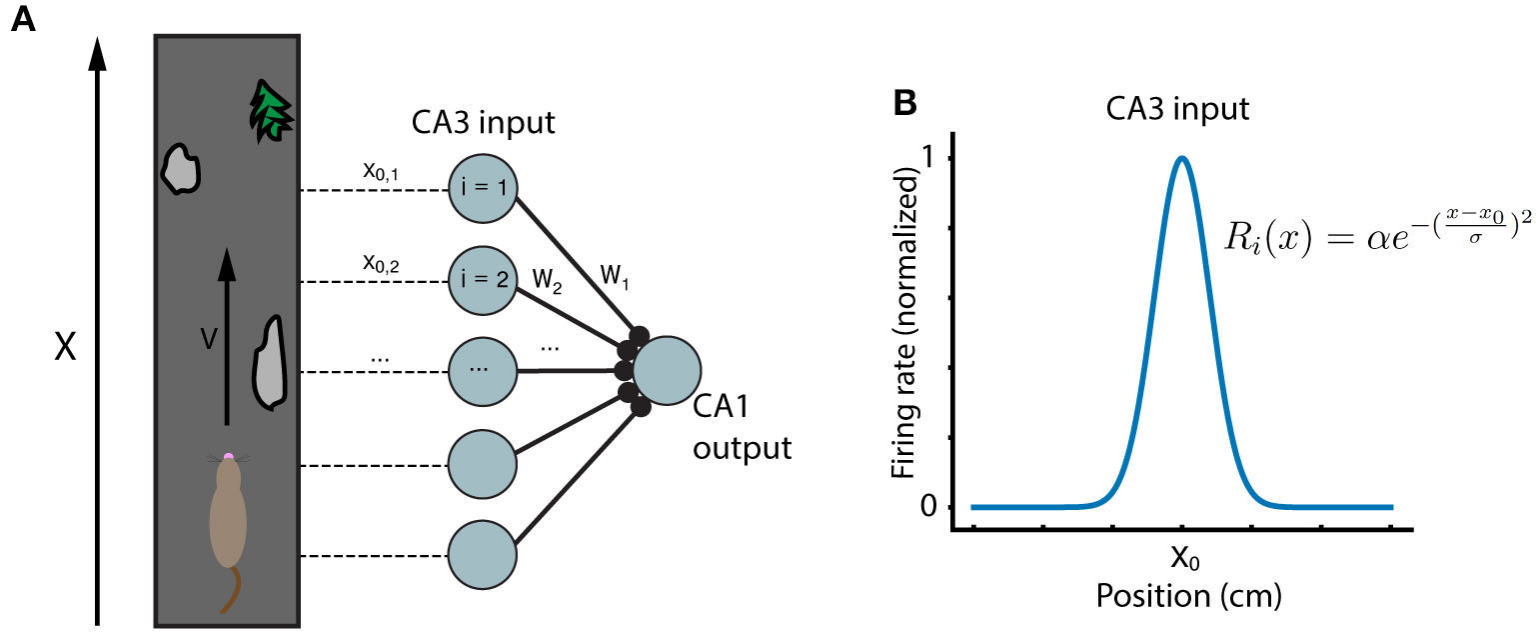
Running track and model network. **(A)** A mouse runs at velocity v along a running track with locations marked by unique features. Inside the mouse hippocampus, N CA3 place cells have activity peaks at different locations along the track, and synapse onto a single postsynaptic CA1 cell. **(B)** The CA3 place cells considered here are modeled as simple Gaussians centered at evenly spaced locations along the running track.

The general framework for the model emulates the setup of recent experiments in hippocampus (Bittner et al., 2015, 2017; Milstein et al., 2020). A mouse runs along a treadmill of length L at velocity *v*. Experimenters record from a postsynaptic CA1 place cell which receives inputs from N presynaptic CA3 inputs. As the animal runs along the track, a dendritic calcium spike (“plateau potential”) is artificially triggered in the CA1 cell, at the same location each lap, in order to induce learning. The CA3 inputs are themselves place fields which tile the length L of the running track (Figure 1). The firing rate *R*_*i*_ of each CA3 input *i* is modeled as a Gaussian function of position:

(1) $$\begin{array}{l} R_{i}\left(x\right)=\alpha e^{-{\left(\frac{x-x_{0}}{\sigma }\right)}^{2}} \end{array}$$

Where *x*_0_ is the center of the given input receptive field. The CA1 output has a ramp potential (i.e., the membrane potential relative to rest, low-pass filtered to eliminate spikes, see (Milstein et al., 2020) determined by the sum of its synaptic input:

(2) $$\begin{array}{l} V\left(x\right)=\beta \overset{N}{\underset{i=1}{\sum }}W_{i}R_{i}\left(x\right) \end{array}$$

Each synapse in our model produces two traces, one for LTP and one for LTD, upon presynaptic firing *R*_*i*_. The equation for each trace $\left(T_{i}^{k}\right)$ has the form:

(3) $$\begin{array}{l} \frac{dT_{i}^{k}}{dt}=\left[-\left(T_{i}^{k}-T_{0}^{k}\right)+\eta^{k}R_{i}\left(v\cdot t\right)\left(T_{max}^{k}-T_{i}^{k}\right)\right]/\tau^{k} \end{array}$$

where $T_{i}^{k}$ is a trace for synapse *i*, *k* indicates either LTP or LTD, $T_{0}^{k}$ is the basal value of the trace for that synapse, $T_{max}^{k}$ is the maximal value of the trace, τ^*k*^ the time constant, and η^*k*^, an activation rate constant. By a simple change of variables *T*_*i*_ → (*T*_*i*_ + *T*_0_), and *T*_*max*_ → *T*_*max*_ + *T*_0_, one gets the slightly simpler equation:

(4) $$\begin{array}{l} \frac{dT_{i}^{k}}{dt}=\left[-T_{i}^{k}+\eta^{k}R_{i}\left(v\cdot t\right)\left(T_{max}^{k}-T_{i}^{k}\right)\right]/\tau^{k}. \end{array}$$

**Figure 2:**
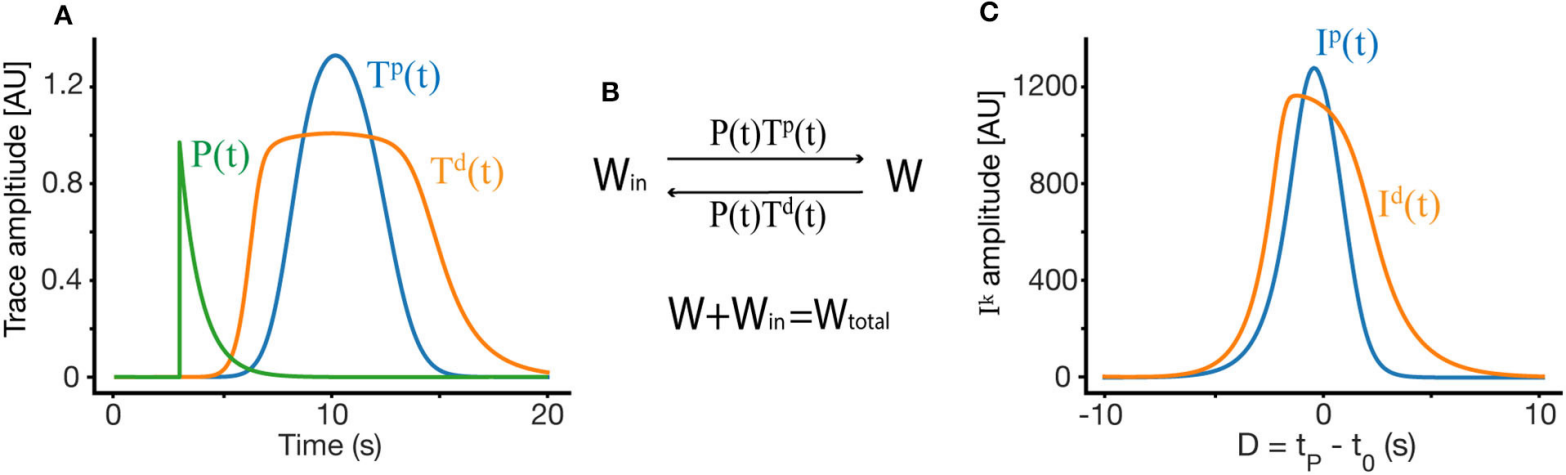
Weight dynamics. **(A)** An illustration of the synaptic plasticity traces and the instructive signal. **(B)** A simple dynamical model of synaptic plasticity, where both LTP and LTD require an overlap between a trace variable *T*^*k*^ and an instructive signal *P*. *W* is the active synaptic weight, and *W*_*in*_ are internalized resources, the total synaptic weight is conserved. **(C)** The overlap *I*^*k*^ between the traces and the instructive signal, as a function of D, where *D* = *t*_*P*_ − *t*_0_ is the displacement between the start of the instructive signal and the center of the presynaptic place field in units of time.

These traces act as transient markers of the presynaptic firing history, allowing the network to bridge events that occur within the temporal scale of the trace (τ^*k*^). The ODE dictating the traces has two terms, the first of which is a decay term—in the absence of presynaptic firing, this term causes the traces return to their basal level at a rate determined by the time constant τ^*k*^. The second term is an activation term, wherein presynaptic firing causes the traces to approach their saturation value $T_{max}^{k}$. The shape of the trace depends on the trace parameters and on the shape and location of the place field of the presynaptic neuron to synapse *i*. Some examples of such traces can be found in Figure 2A.

For a specific functional shape of an input place field *R*(*x*), the place field in time has the form *R*(*v* · *t* − *x*_0_) = *R*(*v* · (*t* − *t*_0_)) where *x*_0_ indicates the place field center in space. Therefore, traces too can be written as $T_{i}^{k}\left(t-t_{0}\right)$. The traces interact with an “instructive signal” *P*(*t*) which is triggered by the induced plateau potential:

(5) $$P(t-t_{P})=\{\begin{array}{cc} 0: & t<t_{P}\\ \gamma e^{-\frac{t-t_{P}}{\tau_{I}}}: & t\geq t_{P} \end{array}$$

Where *t*_*P*_ marks the time of induction of the plateau potential. This instructive signal is global, acting across all synapses in tandem with the synapse specific traces.

Our learning rule is a very simple induction model, which depends on above described synaptic traces $T_{i}^{k}$, instructive signal *P*(*t*), and assumes a conserved resource that can become synaptic efficacy (see Figure 2B). These assumptions produce a simple ODE for the dynamics of synaptic plasticity:

(6) $$\begin{array}{l} \frac{dW_{i}}{dt}=\left(1-W_{i}\right)\cdot T_{i}^{p}\left(t-t_{0}\right)P\left(t-t_{P}\right)\\ \;-W_{i}\cdot T_{i}^{d}\left(t-t_{0}\right)P\left(t-t_{P}\right) \end{array}$$

Where again we denote the start time of the instructive signal as *t*_*P*_ and the temporal center of the presynaptic place field as *t*_0_. Here we set *W*_*total*_ from Figure 2B to 1, such that *W*_*in*_ is then equal to 1−*W*. Note that the assumption of a conserved quantity results in a plasticity rule that is weight dependent (Milstein et al., 2020). By changing variables such that *t* → *t* + *t*_0_ we get:

(7) $$\begin{array}{l} \frac{dW_{i}}{dt}=\left(1-W_{i}\right)\cdot T_{i}^{p}\left(t\right)P\left(t-D\right)-W_{i}\cdot T_{i}^{d}\left(t\right)P\left(t-D\right) \end{array}$$

where *D* = *t*_*P*_ − *t*_0_ is the displacement between the start of the instructive signal and the center of the presynaptic place field in units of time. These presynaptic place fields are tiled along the length L of the running track, therefore for each synapse *i*, *D* is different, but simply linearly shifted. If the set of presynaptic neurons have centers that are equally and linearly spaced with a spacing Δ*x*, starting at *x*_0_ then *t*_0_(*i*) = *t*_*P*_ − (*x*_0_(*i*) + *i* · Δ *x*)/*v*), where *t*_0_(*i*) is the center in time or the presynaptic receptive field of synapse *i*. Similarly one could write *D* = *D*(*i*), where each version of *D* has he same order but is simply shifted.

### 2.2. General Solution and Fixed Point

To find the fixed point solution of our learning rule, we can integrate over a single trial and assume that during that trial, *W* does not change significantly, so it can be taken out of the integral. This approximation is appropriate even during fast learning when *W* reaches a fixed-point. The dynamics will therefore depend on the integral of the overlap between the traces and the instructive signal (Figure 2C):

(8) $$\begin{array}{l} I_{i}^{k}\left(D\right)=\int_{0}^{t_{trial}}T_{i}^{k}\left(t\right)P\left(t-D\right)dt \end{array}$$

where *k* ∈ (*p, d*). The fixed point of Equation (6) is therefore:

(9) $$\begin{array}{l} \left(1-W_{i}\right)\cdot I_{i}^{p}\left(D\right)=W_{i}\cdot I_{i}^{d}\left(D\right) \end{array}$$

This implies that the fixed point of *W*_*i*_ is simply:

(10) $$\begin{array}{l} W_{i}\left(D\right)=\frac{I_{i}^{p}\left(D\right)}{I_{i}^{p}\left(D\right)+I_{i}^{d}\left(D\right)} \end{array}$$

Practically speaking, this fixed point *W*_*i*_(*D*) gives us the final weights of all the synapses in response to an instructive signal presented at time *t*_*P*_ as a function of their temporal distance to this instructive signal, *D* = *t*_*P*_ − *t*_0_. This fixed point can be calculated numerically in the general case, but can also be calculated analytically under certain conditions (see section 2). The fixed point can also be described in terms of spatial dependence, the units for which can be obtained in the case of constant velocity simply by multiplying *D* by the animal's velocity *v*. The fixed point weights can be converted to the fixed point ramp amplitudes through Equation (2).

Equation (10) implies that with linear induction of traces, as assumed in this section, the parameters of the two traces must be different if we want the weights to depend on the displacement between the place field center and the instructive signal. If the traces are identical for all *D*, *W*_*i*_(*D*) = 0.5 for all *D*. If they have the same functional form, but a different scale such that *I*^*p*^ = *k*·*I*^*d*^ then *W*_*i*_(*D*) = *k*/(1 + *k*) for every *D*. For the formulation of traces in Equation (3), this would occur, for example, if the parameters η^*d*^ = η^*p*^ and τ_*d*_ = τ_*p*_ but $T_{max}^{p}\neq T_{max}^{d}$. In order for the weights to produce place fields that are selective for a certain range of D, the overlap *I*^*d*^ should generally be broader and shallower than the overlap *I*^*p*^. This can be implemented directly by choosing appropriately different trace parameters for LTD and LTP, and/or by including a basal level of LTD $T_{0}^{d}$.

We can find a closed form solution for the traces by directly integrating Equation (3), which is a non-homogeneous linear first order ODE and therefore can be solved using an integration factor *U*(*t*). Where:

(11) $$\begin{array}{l} U\left(t\right)=e^{\frac{1}{\tau }\int_{-\infty }^{t}\left(1+\eta R_{i}\left(v\cdot t^{\prime }\right)\right)dt^{\prime }} \end{array}$$

The solution for Equation (3) then has the form:

(12) $$\begin{array}{l} T_{i}\left(t\right)=\frac{T_{max}}{\tau }\frac{\left[\int_{-\infty }^{t}dt^{\prime }\eta R_{i}\left(v\cdot t^{\prime }\right)U\left(t^{\prime }\right)+C\right]}{U\left(t\right)} \end{array}$$

Given $R_{i}\left(v\cdot t^{\prime }\right)$, one can use Equation (12) to solve for *T*_*i*_(*t*), and therefore $I_{i}^{k}$, and therefore *W*_*i*_. Some choices of $R_{i}\left(v\cdot t^{\prime }\right)$ are more analytically tractable than others, hence the choice of rectangular presynaptic place fields (Equation 18) for our full analysis (see section 2.8 and Appendix), as they allow for a closed form solution to this equation.

### 2.3. Linear Track

To investigate the evolution of the weights and their convergence to fixed points, we first consider the case of a linear track. For a linear track, we assume after each lap the animal “restarts,” such that for a single trial, previous traversals of the track do not interfere. By calculating Equation (10) for a set input receptive field shape *R* and an instructive signal *P*, we can numerically solve for the fixed points. The resulting steady state place field shapes depend on the different parameters of the LTP and LTD traces.

**Figure 3:**
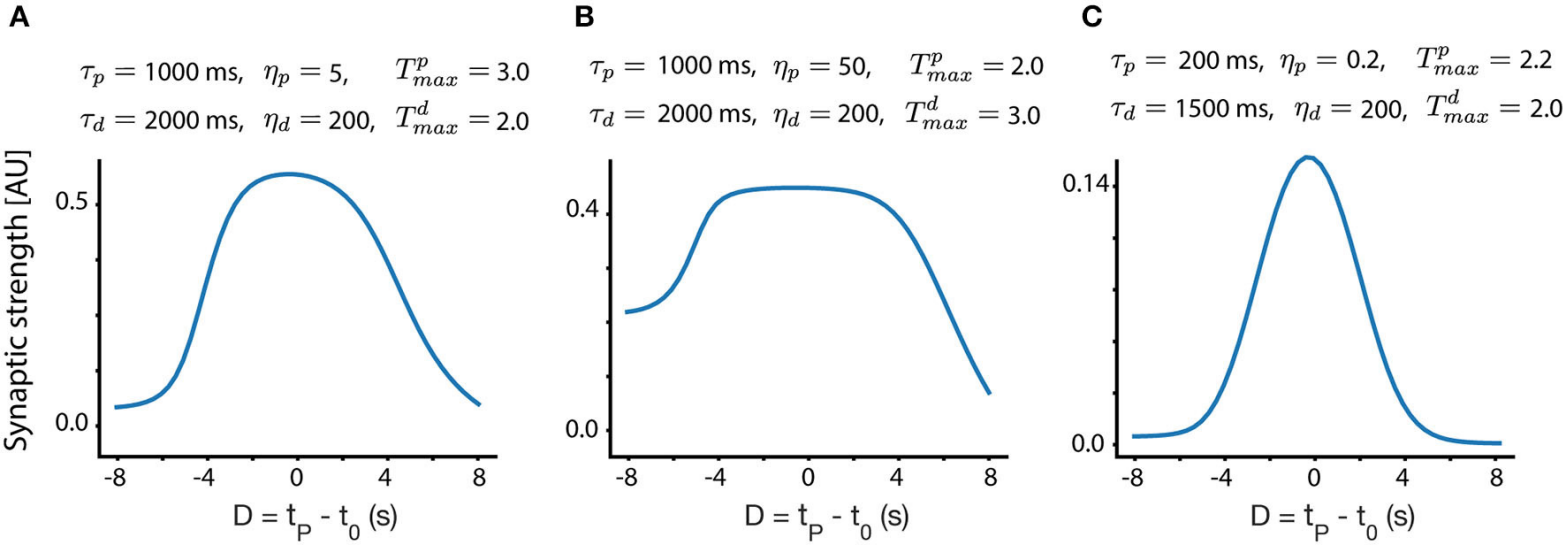
Fixed point place field structure. **(A–C)** Fixed point weights as a function of D for different sets of parameters. The velocity in all these subplots is identical (*v* = 0.116 m/s, chosen to match the velocity in Milstein et al., 2020), and the same presynaptic Gaussian place field is used, with a SD width of 0.21 m. All other parameters are indicated above each subplot.

In Figure 3, we show different place field fixed points, with the same presynaptic place field and the same velocity, but with different trace parameters, as indicated above each subplot. One can observe that the width, selectivity, symmetry and overall shape of the place fields significantly depends on these parameters. From experiments (Bittner et al., 2015, 2017; Milstein et al., 2020) we know that the shape of the fixed point should be such that it is maximal near *D* = 0 and gets smaller, approximately symmetrically as *D* deviates from zero. For a large enough |*D*|, *W*_*i*_ should be close to zero. Given these experimental observations, one can infer different trace and instructive signal parameters that are consistent with experiments.

**Figure 4:**
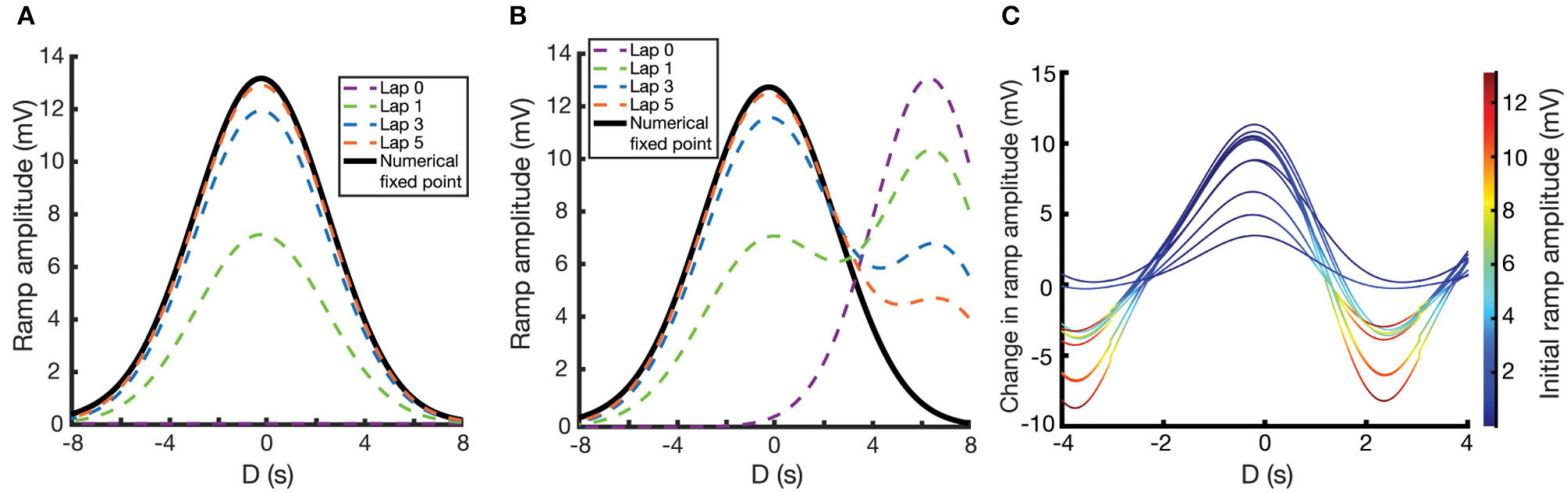
Place field plasticity on a linear track. **(A,B)** Simulated evolution of ramp amplitude over laps (dashed lines) and the numerically calculated fixed point ramp amplitude (solid lines) as a function of D. For **(A)**, initial weights are set to zero. For **(B)**, weights are initialized such that there is a preexisting place field at *D* = 7.0 s. **(C)** Change in ramp amplitude as a function of D and the initial ramp amplitude, for 10 different simulations. To replicate experiment, each simulation has a random selection of initial place field strength, plateau potential strength, and plateau potential location. These response curves replicate the results of Figure 1J of Milstein et al. (2020), with the caveat that our response curves are smoother and more uniform since we are assuming a uniform velocity.

These numerical calculations can then be verified by performing simulations where *W*_*i*_ is explicitly updated at every time-step using Equation (7). For the following simulations, we use the same parameters as in Figure 3C. We examine here two different cases. In the first case, initial weights are set to zero, and so the weights smoothly converge to their unimodal fixed point as the mouse repeats laps along the track. Figure 4A shows the fixed point CA1 output ramp amplitude (Equation 2) that results from this case. Notice that the shape of the fixed point ramp amplitude (Figure 4A) is very similar to the shape of the fixed point weights (Figure 3C), owing to the fact that the inputs *R*_*i*_(*x*) in Equation (2) are themselves Gaussian. The emergence of a place field is an example of effective one-shot learning in this case, as it emerges and is well-defined after the first lap. The subsequent laps asymptotically converge that place field to its final fixed point.

In the second case (Figure 4B), the weights are initialized such that there is a preexisting place field in the CA1 output. Over the course of learning, interaction between the traces and the instructive signal cause the new place field (centered at the location of the instructive signal, *D* = 0) to potentiate and the preexisting place field to depress. The further away the initial place field is from the new place field, the longer it will take the initial place field to depress, owing to a smaller overlap (*I*_*k*_) between the traces and the instructive signal. However, the fixed point of the weights are irrespective of the initial place field, so given enough laps, the initial place field will always disappear, and the solution will become unimodal.

Note that in both of these cases, and despite the assumptions made in deriving the fixed point solution of Equation (10), the simulated results (dashed lines in Figure 4) converge to the numerical results (solid lines in Figure 4).

Running multiple simulations, each with their own random initial place field amplitudes and instructive signal locations/amplitudes, one can plot the change in place field ramp amplitude as a function of initial place field ramp amplitude and the time relative to plateau onset. These resulting response curves (each one representing a different simulation and different instantiation of these random parameters) predict the change in ramp amplitude following the induction of a plateau potential, in a given location and with a given initial ramp voltage (Figure 4C). These curves replicate the results of Figure 1J of Milstein et al. (2020), although our use of a fixed velocity makes our curves appear smoother and more uniform.

### 2.4. Circular Track

A similar exercise can be performed assuming a circular track with continuous running, where the instructive signal/traces can span across laps and place fields are periodic. We can think of each cycle as another iteration in which the final value of traces on the previous run is their initial value of the current run. In this case it is more complicated to calculate the numerical fixed point, but one way to do so is to consider an infinite number of iterations, and use the convergence of the infinite series.

For a circular track, we must account for the wraparound of both the traces and the instructive signal in our calculations. If we define as *t*_*max*_ as the time it takes to do one cycle, then the total time up to time *t* can be rewritten as *t* = *n*·*t*_*max*_+*q*, where *n* is the number of fully completed cycles. On the *n*+1 run, the initial condition denoted by *C*_*n*_ is *T*_*i*_(*n*·*t*_*max*_). Using these definitions, and the periodicity of the place cell with a period *t*_*max*_, the solution for Equation (12) is:

(13) $$\begin{array}{l} {\tilde{T}}_{i}^{k}\left(t\right)=\frac{T_{max}^{k}}{\tau }\left[\int_{n\cdot t_{max}}^{n\cdot t_{max}+q}dq^{\prime }\eta^{k}R_{i}\left(v\cdot q^{\prime }\right)U^{k}\left(q\right)\right]/U^{k}\left(q\right)\\ \;+T_{i}\left(n\cdot t_{max}\right)/U^{k}\left(q\right) \end{array}$$

where the index *k* is *p* or *d* for LTP and LTD traces, respectively. The first part on the right is the same for *q* in each period, and independent of *T*_*max*_. The second part iteratively forms a series such that: ${\tilde{T}}_{i}^{k}\left(n\cdot t_{max}\right)=T_{i}^{k}\left(t_{max}\right)\overset{n-1}{\underset{j=0}{\sum }}{\left(U^{k}\left(t_{max}\right)\right)}^{-j}$ which in the infinite limit converges to: $T_{i}\left(t_{max}\right)/\left(1-U{\left(t_{max}\right)}^{-1}\right)$ This implies that the solution for the circular track converges to:

(14) $$\begin{array}{l} {\tilde{T}}_{i}^{k}\left(q\right)=\frac{T_{max}^{k}}{\tau }\left[\int_{0}^{q}dq^{\prime }R_{i}\left(v\cdot q^{\prime }\right)U^{k}\left(q\prime \right)\right]/U^{k}\left(q\right)\\ \;+T_{i}\left(t_{max}\right)/\left(1-U{\left(t_{max}\right)}^{-1}\right) \end{array}$$

and $T_{i}^{k}\left(t_{max}\right)=\frac{T_{max}}{\tau }\left[\int_{0}^{t_{max}}dqR_{i}\left(v\cdot q\right)U^{k}\left(q\right)\right]/U^{k}\left(t_{max}\right)$

**Figure 5:**
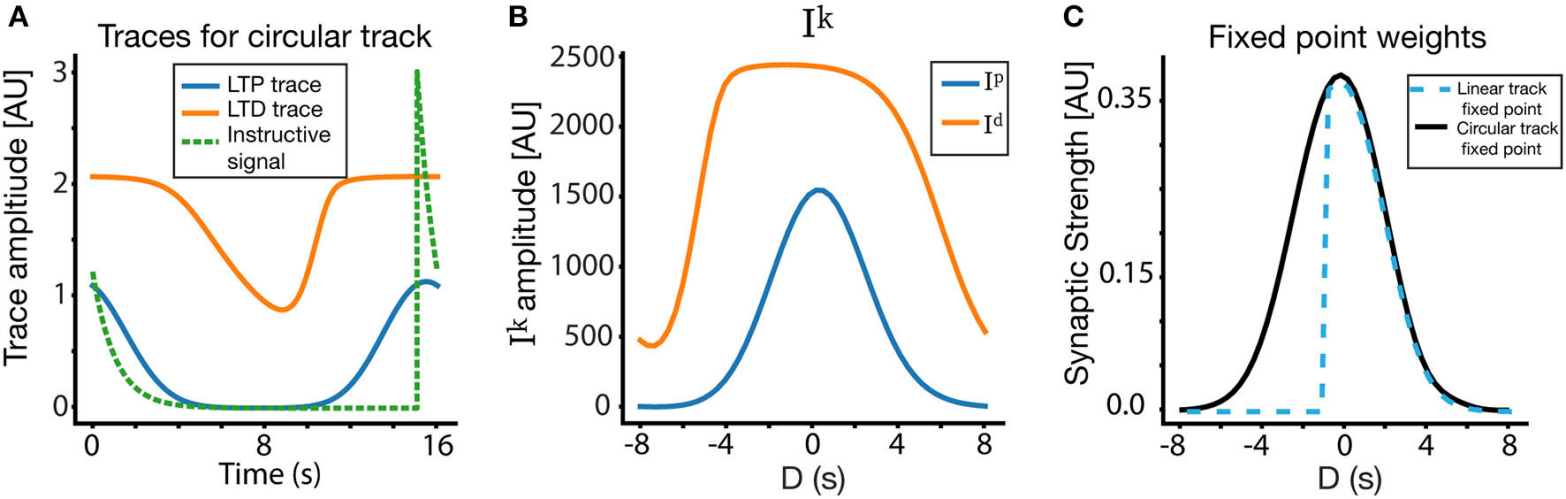
Place field plasticity on a circular track. **(A)** Traces and instructive signal for a lap >> 1 on the circular track, as a function of time. The presynaptic traces plotted are from a CA3 input centered at location of the instructive signal. **(B)** *I*_*p*_ and *I*_*d*_ on circular track. **(C)** Fixed point for weights on the circular track, compared to fixed point weights for the linear track. Notice also that **(A)** is plotted in absolute time, where as **(B,C)** are plotted in terms of *D* (time relative to instructive signal onset). The solution in the circular track is nearly symmetric around *D* = 0, while the linear solution contains goes to zero for *D* < −0.5*s* due to its lack of periodic boundary conditions.

In order to illustrate this graphically, we show in Figure 5A the traces for a lap >> 1, where due to the periodicity of the track, the traces' (and the instructive signal's) values at the end of the track are equal to those at the beginning of the track (periodic boundary conditions). In the linear case, both the traces and instructive signal are reset to zero at the beginning of the track. For the example in Figure 5, we place the instructive signal near the edge of the track, so as to accentuate the difference between the circular and linear case. The traces in Figure 5A are also chosen from a presynaptic cell centered at the location of the instructive signal. The overlaps *I*_*k*_ (Figure 5B) between the traces and the instructive signal are then used to calculate the fixed point. Figure 5C compares the fixed point weights in both the linear and circular case, using the same parameters and inputs. Notice that, due to the lack of periodic boundary conditions, the linear track fixed point is 0 for *D* < −0.5*s* (since the instructive signal and presynaptic place fields do not have PBC, there are no presynaptic place fields whose centers are more than 0.5 s ahead of the instructive signal), while the circular track produces near symmetrical solutions. It must be noted that for many cases and a wide range of parameters, the circular track solution closely matches the linear track solution. In particular, if the time constant of the traces is shorter than the remaining time of track traversal, the traces will decay before the track ends, meaning that the traces will be at or near zero at the start of the next lap in both the linear and circular case. As a result, the biggest differences between the circular and linear track fixed points occur for traces/instructive signals near the boundaries of the track, and/or when the trace time constants are long relative to the traversal time of the track.

### 2.5. Non-linear Trace Activation

A further modification can be made to the circular track model by assuming a non-linear response of the traces to presynaptic firing. The assumption of non-linear activation is used in the model of Milstein et al. (2020), but is not necessary to model BTSP. Our model forgoes the need for this non-linearity, allowing for simpler equations and improved analytical tractability. Still, here we present an example of our model with the assumption of non-linear traces to show such an assumption is also compatible. Here we assume that instead of the presynaptic firing rate *R*, directly activating the traces, this activation is non-linearly filtered though a function *F*^*k*^(*R*) where the index *k* can take the values *p* or *d* indicating a possibly different non-linearity for LTP and LTD traces. Thus the trace activation equation now takes the form:

(15) $$\begin{array}{l} \frac{dT_{i}^{k}}{dt}=\left[-\left(T_{i}^{k}-T_{0}^{k}\right)+\eta^{k}F^{k}\left(R_{i}\left(v\cdot t\right)\right)\left(T_{max}^{k}-T_{i}^{k}\right)\right]/\tau^{k}. \end{array}$$

**Figure 6:**
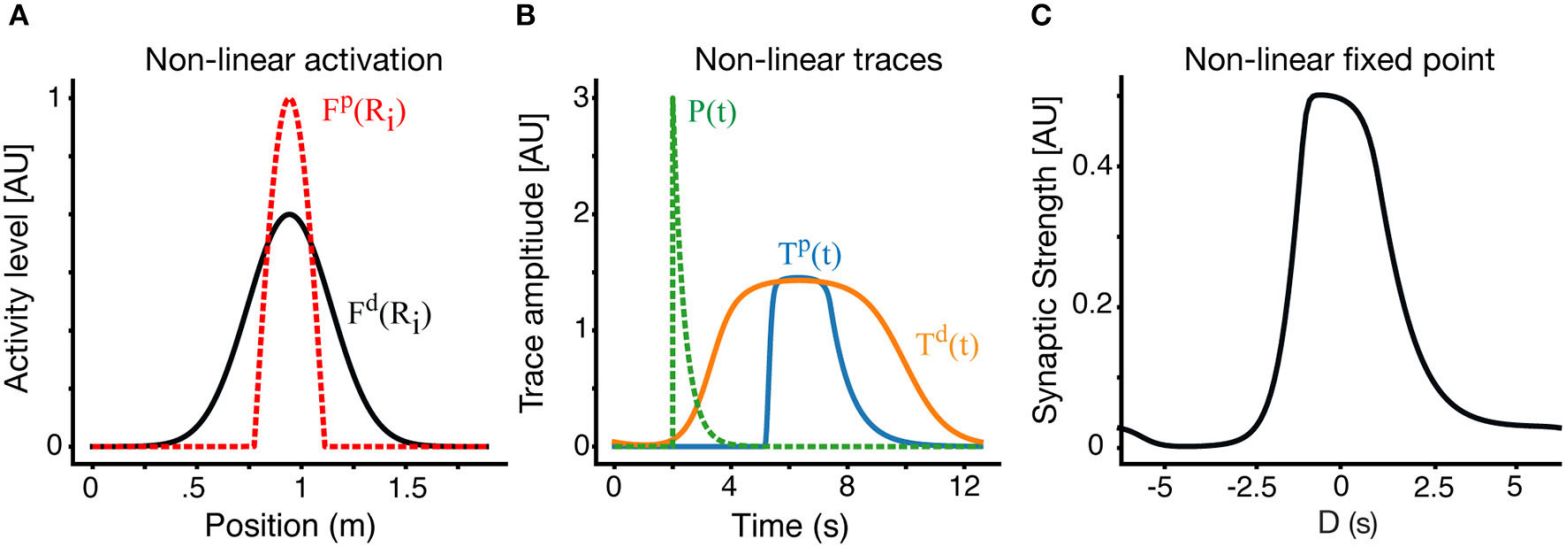
Place fields with non-linear traces. **(A)** In an example non-linear case, the effective presynaptic activity profiles that drive LTP and LTD traces are different. Here the LTD traces are driven by the linear RF as above (black) but LTP traces are driven by a non-linear modification of the RF (dashed-red). **(B)** LTP (blue) and LTD (orange) traces in the non-linear case. The instructive signal in dashed green. **(C)** The resulting fixed point of the weight vector. Results here are for *V* = 0.15 *m*/*s*.

For simplicity we have chosen a thresholded linear form: $F^{k}\left(x\right)=C^{k}{\left[x-\theta^{k}\right]}_{+}$. Practically it implies that the LTP and LTD traces see different effective presynaptic receptive fields, and aside from that, all the previous analysis still holds. Solutions with non-linear traces on a circular track are shown in Figure 6. In Figure 6A, we show the effective receptive fields for the LTP trace (dashed red) and LTD trace (black) respectively. Apart from the non-linearity, in this example, the parameters of the LTP and LTD traces are identical. The narrower LTP effective place field generates a narrower LTP trace, which allows for a selective receptive field (Figure 6C), despite the parameters of both traces being the same.

### 2.6. Velocity Dependence

**Figure 7:**
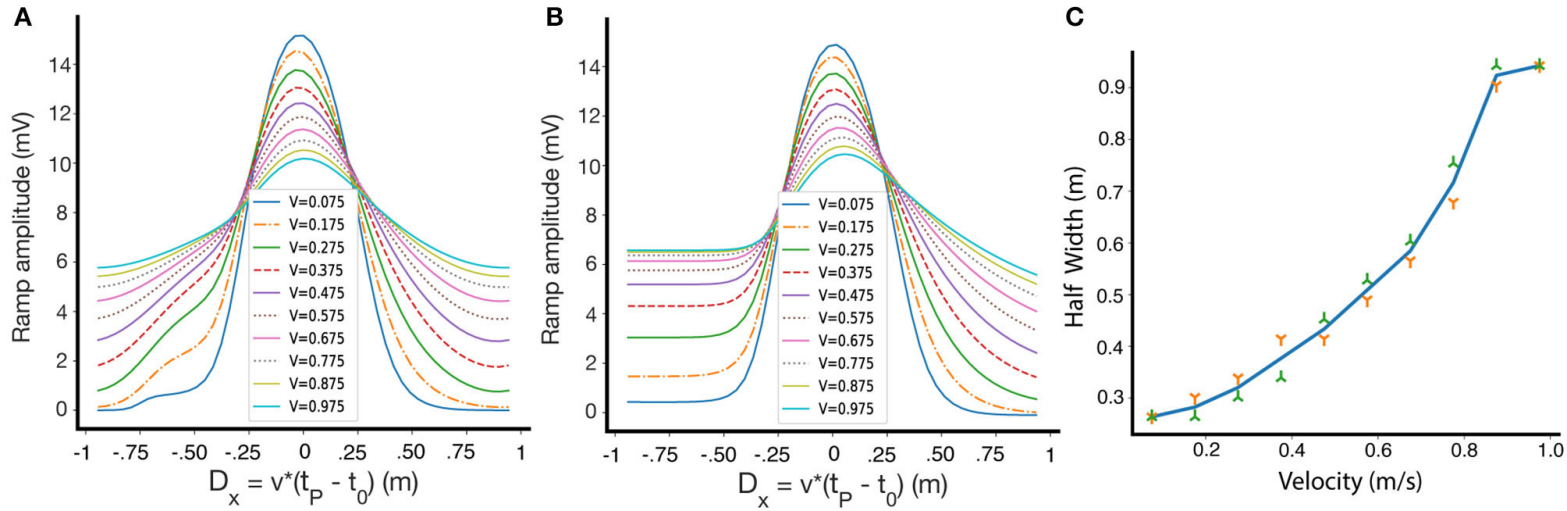
The dependence of place field shape on velocity during induction. **(A)** Fixed points of the ramp amplitude as a function of *D*_*x*_ for differing movement velocities, circular track. Inset: velocities during induction, in m/s. **(B)** Same as **(A)**, but for a linear track. The lack of periodic boundary conditions introduces some asymmetry in the fixed point ramp amplitudes. **(C)** The dependence of the half width of the weights at fixed-point on the travel velocity during induction along a circular track. The green and orange symbols are for left and right widths, respectively.

Until now, we have only shown results for one fixed running speed, but the shape of the resulting place fields depends on the velocity of the animal on the track during the induction phase. Figure 7A shows how the shape and selectivity of place fields depends on velocity, for a given set of parameters on a circular track. We also demonstrate the velocity dependence for a linear track in Figure 7B. In Figure 7C we show that for a circular track, the width of the place field monotonically increases, within a large velocity range, as the velocity of the animal increases. These results are consistent with experimental results, which show that as the velocity increases, the width of the resulting place field increases and its selectivity decreases (Bittner et al., 2017). However, our model also predicts that the amplitude of the learned place fields decreases as a function of velocity. Qualitatively similar results are obtained for other parameter sets, and for non-linear traces. After induction, in the absence of an instructive signal, the place fields retain their size and are independent of velocity.

Note that the fixed point of W here is plotted against *D*_*x*_ = *v*(*t*_*p*_ − *t*_0_), (which is simply D multiplied by the velocity), so that we can compare the resulting place fields at the same positions. While at every velocity there is a single fixed point curve, experimentally we might not reach the fixed points because the convergence time might be large.

### 2.7. Convergence Time

**Figure 8:**
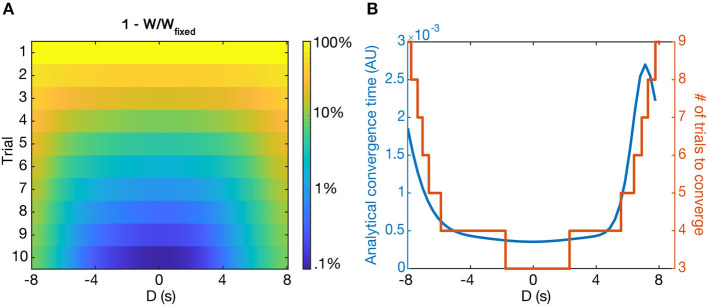
Convergence to solutions. **(A)** The relative distance to the fixed point ($\frac{W_{fixed}-W}{W_{fixed}}$) as a function of *D* and trial number, shown as logarithmic heatmap. **(B)** The convergence time, τ_*w*_ (blue), and the number of simulated trials to reach $\frac{W_{fixed}}{e}$ away from the fixed point (orange) as a function of *D* (in seconds). Notably, in both cases the convergence time rises steeply beyond a certain value of D. Both cases start from the initial condition *W*_*i*_ = 0.

The fixed point of the learning rule produces a single place field centered at the location of the induced plateau potential, regardless of pre-existing place fields before learning. However, our model predicts that the rate at which learning converges depends on D—the larger absolute value of D, the longer the convergence time. This effect is present for both the potentiation of new place fields and the depression of old place fields. Figure 8A shows the simulated convergence of the weights toward their fixed points as a function of trial number and D, given zero initial weights.

To predict the convergence time analytically, we use the approximation that W does not change significantly during a single trial, and take the integral over a single trial as in Equation (8) in order to rewrite the dynamics of Equation (6) over trials as:

(16) $$\begin{array}{l} \delta W=I_{p}-W\left(I_{p}+I_{d}\right) \end{array}$$

where *I*_*p*_ and *I*_*d*_ are defined as in Equation (8), and the notation δ is used instead of the temporal derivative to indicate that this is the change over the whole trial.

Explicitly, the solution to Equation (16) is:

(17) $$\begin{array}{l} W\left(t\right)=\frac{I_{p}}{I_{p}+I_{d}}\left(1-e^{-\left(I_{p}+I_{d}\right)t}\right) \end{array}$$

Though the assumption made in order to reach this equation (that W does not change much within each trial) is not guaranteed to be a good approximation in all cases, the weights do change slowly within each trial (a) when they are close to their fixed point, and (b), when they are far from *D* = 0. Therefore, in these cases Equation (17) is a reasonable estimate of the weight dynamics, and $\tau_{w}=\frac{1}{I_{p}+I_{d}}$ is a good approximation of a convergence time constant.

Simulations show that the analytically predicted convergence time is closely related to the number of trials it actually takes for the simulated weights to reach $\frac{1}{e}$ away from the fixed point value of $\frac{I_{p}}{I_{p}+I_{d}}$ (Figure 8B) when starting from zero initial weights. Notably, the convergence time steeply rises for large absolute D, such that it takes around 3–5 times as many trials to converge for |*D* = 8| s as it does for *D* = 0 s. For even larger D, τ_*w*_ can become prohibitively long—we predict that the output could effectively maintain two place fields for long periods of time, provided they are far enough apart from each other, as the old place field will depress incredibly slowly.

### 2.8. Analytical Solution for Rectangular Place Fields

The numerical integrals needed to find the traces $T_{i}^{k}$, and their overlaps with the instructive signals, $I_{i}^{k}$ can in some cases be solved analytically. A simple example is when presynaptic receptive fields are rectangular. We examine that case here, assuming that the input place fields start at *t*_1_ and end at *t*_2_, with an amplitude of α. In terms of the position variable these start at *vt*_1_ and end at *vt*_2_. Formally the place field has the form:

(18) $$R(t)=\{\begin{array}{cc} 0: & \;t\leq t_{1}\\ \alpha : & t_{1}<t<t_{2}\\ 0: & \;t\geq t_{2} \end{array}$$

**Figure 9:**
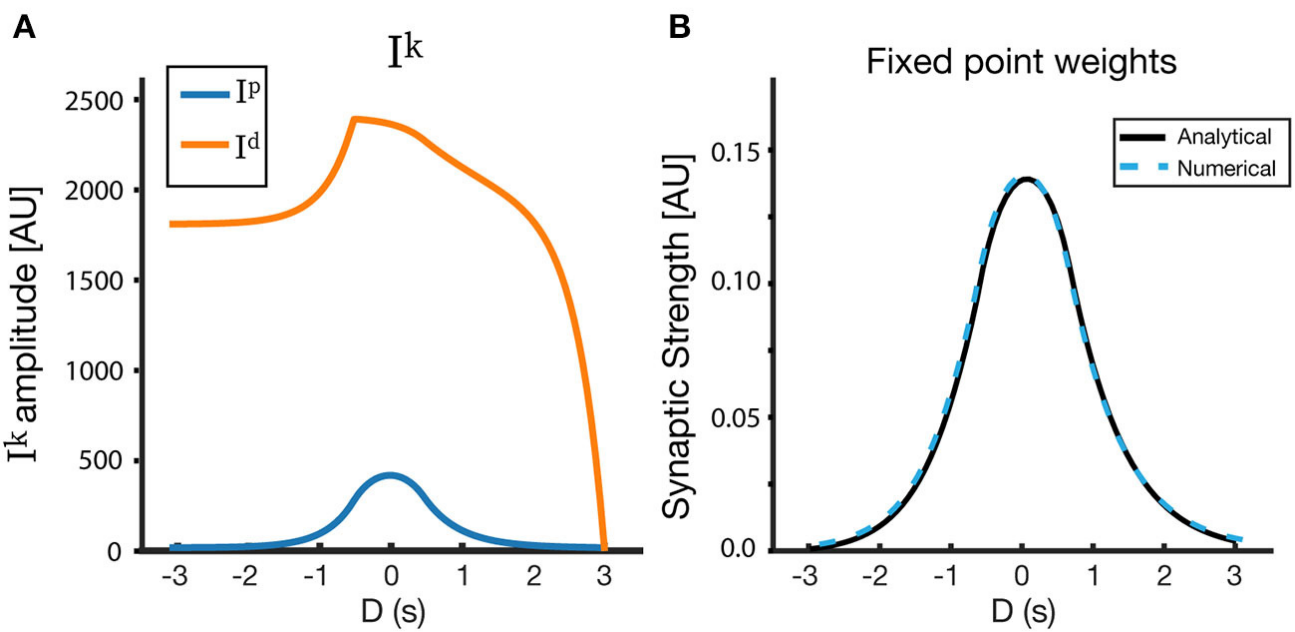
Analytical solutions for a rectangular receptive field. **(A)** *I*_*p*_, *I*_*d*_, and **(B)** *W*_*fixed*_ as a function of *D*. Here, the LTD trace has a basal level $T_{0}^{d}$, which creates an additional overlap $\gamma T_{0}^{d}\tau_{I}\left(1-e^{\frac{t_{P}-t_{t}rial}{\tau_{I}}}\right)$ (see Appendix). The resulting fixed point is nearly symmetrical around *D* = 0, and its properties can be modified via the model parameters. The track is linear, and the parameters used for the figure are as follows: τ_*p*_ = 500 ms, τ_*d*_ = 1,500 ms, η_*p*_ = 0.25, η_*d*_ = 200, $T_{max}^{p}$ = 2.2, $T_{max}^{d}$ = 2.0, $T_{0}^{p}$ = 0, $T_{0}^{d}$ = 1.5.

Using this simple form of *R*(*t*), we can explicitly solve $T_{i}^{k}$ and therefore $I_{i}^{k}\left(D\right)$ (Equation 8), in the case of the linear track. By performing these calculations (see Appendix), we arrive at the fixed point $W_{i}=\frac{I_{i}^{p}}{I_{i}^{p}+I_{i}^{d}}$. The final analytical form of the fixed point depends on the activation rate (η), saturation values ($T_{max}^{k}$) and time constants (τ^*k*^) of the traces, the location (*t*_1_ and *t*_2_) and amplitude (α) of the receptive field, location (*t*_0_) and time constant (τ_*p*_) of the instructive signal, and on the duration of the lap *t*_*trial*_. We also include in this analysis a basal level of LTD ($T_{0}^{d}$), which smooths over some of the artifacts of the rectangular input place field, and makes the solution more similar to the solutions with Gaussian input place fields demonstrated above. Figure 9 shows the results of the analytical solution. The fixed points found analytically match those found via simulations (and those found via numerical methods), given the same input PF and same parameters are used in both cases (Figure 9B).

## 3. Discussion

Synaptic plasticity that operates on behavioral time scales has been shown to determine place field plasticity in CA1 neurons. The underlying structure of such plasticity is significantly different than the commonly studied Hebbian forms of plasticity that assume near temporal coincidence of pre- and postsynaptic activity. Experimental results in CA1 using both *in vivo* and slice preparations strongly suggest that presynaptic activity generates synaptic eligibility traces for both LTP and LTD (Bittner et al., 2017; McKenzie et al., 2019). Prior modeling work (Milstein et al., 2020) shows, using simulations, that these two opposing traces, combined with a weight dependence can account for the place field plasticity observed *in vivo*.

Here we proposed a simple formulation for two-factor plasticity (Frrémaux and Gerstner, 2016) that depends on eligibility traces for both LTP and LTD, an instructive signal and weight dependence. We have shown that this rule can be mathematically analyzed to yield a simple expression for the fixed points of these place fields. These results show that place fields can have spatial selectivity only if the traces have essentially different temporal dynamics, or if they are induced non-linearly, and with different non-linearities. Such results are general, beyond the specific shapes of presynaptic place fields and the model's parameters. The general rule in both cases is that for obtaining selective place fields, the overlap between the LTD trace and the instructive signal should have a broader shape than the overlap between the LTP trace and the instructive signal; the more pronounced this difference, the sharper the place field. By assuming specific shapes of presynaptic activity and of the instructive signal, we can calculate exactly the shape of the learned postsynaptic place fields at steady state and can estimate the local convergence rate to these shapes. We also used simulation to validate the analytical results, and predicted that the convergence of place fields is rapid for small D, and slower for larger *D*. The dependence of the convergence rate on the value of *D* is also consistent with the analytical results. In the case of a rectangular presynaptic place field a fully analytical solution is obtained.

The shape of the postsynaptic place field at steady state depends on many of the system parameters and assumptions, such as the shape of the presynaptic place field and the parameters that determine the dynamics of the traces or the instructive signal. One additional parameter that significantly affects the shape of the place field is the animal's velocity during induction. If the animal moves through space slowly, place fields are more local and selective, while a fast velocity results in non-local and broad place fields. However, after induction, when there is no instructive signal, velocity does not effect the size of the place field. The velocity dependence of place field width is a general phenomenon, that does not depend on a narrow set of parameters, and is in agreement with experimental results (Bittner et al., 2017). Our model additionally predicts that place field amplitude will decrease as a function of velocity during induction laps.

We have also predicted that the weakening of prior place fields in old locations will be significantly slower than the strengthening of the previously weak efficacies in the new location. The determining factor for this rate of change is the overlap between both eligibility traces and the instructive signal. Locations near the peak of the instructive signal are the ones that get enhanced, as in such locations there is a maximal overlap between the traces and the instructive signal, and therefore a fast convergence rate. This potentiation convergence rate is effectively one- or two-shot learning (Figure 4A). On the other hand, locations which might have strong initial weights but are far from the instructive signal get weakened. For such signals the overlap between the instructive signal and both traces is much smaller and therefore the convergence to the fixed point is also much slower. This slow depression enables the existence of multimodal place fields for a small number of intermediate trials, which can be observed in Milstein et al. (2020) (see their Figures 1A,G). We predict that such multimodal place fields will be more prevalent, and last for more trials for large |*D*|.

In this formulation we have assumed that the synaptic efficacy dynamics are weight dependent. Such weight dependence can arise from an assumption of a conserved quantity, for example a conserved number of receptors in the membrane and an internal synaptic store. This assumption is also motivated by a previous weight dependent model, that compares well with experimental results (Milstein et al., 2020). The existence of fixed points in our model, and their shape critically depends on this assumption.

In our solution we assume that the animal's velocity is constant throughout, though in a real environment this is not the case. Animals change their running speed as they traverse the track, speeding up and slowing down and even sometimes stopping to eat. Such varying velocity implies that there is no true fixed point with this model, and that the place fields fluctuate around some mean fixed point. For a changing velocity, one can perform simulations using experimentally obtained velocity profiles instead of an analytical solution (Bittner et al., 2017). Such results are more biologically realistic but are much more complex and offer less intuition. It is feasible that using the animal's velocity statistics, the mean around which the place fields fluctuate, the variance with which they fluctuate, etc. can be estimated.

The inclusion of eligibility traces in our learning rule is essential, as it allows the model to associate activity with temporally distal instructive signals and thereby solve the temporal credit assignment problem. Traditional models of learning such as Hebbian plasticity or STDP are insufficient to describe plasticity which occurs on behavioral time scales, as they are restricted to learning tight temporal correlations of activity. Modifications to STDP which attempt to learn associations between stimuli that are seconds apart rely on other factors to bridge this temporal gap, such as having the stimuli themselves decay with a seconds long time constant (Drew and Abbott, 2006). Previous characterizations of eligibility traces have generally depended on both pre- and postsynaptic activity, where the conversion of traces to actual synaptic efficacies depended on a third factor such as a reward signal (Gavornik et al., 2009; Gavornik and Shouval, 2011; Yagishita et al., 2014; He et al., 2015; Frrémaux and Gerstner, 2016; Huertas et al., 2016). In contrast, the model presented here is essentially a two-factor rule, in which traces depend only on presynaptic activity, and weight changes depend on both traces and the instructive signal. Indeed, previous work has shown that experimentally observed behavioral timescale synaptic plasticity in CA1 is in fact inconsistent with three-factor rules which depend on synchronous pre- and postsynaptic activity (Milstein et al., 2020). The observation that place field plasticity in CA1 neurons can be described by a two-factor rule actually makes its analysis simpler, enabling the detailed results found here. In other systems, such as sensory systems (for which previous eligibility trace theories were developed), the aim is to alter the dynamics of a recurrent network, so for such models three-factor might indeed be necessary (Gavornik et al., 2009; He et al., 2015; Huertas et al., 2016). Regardless, both two- and three-factor eligibility trace models provide a simple and mathematically tractable explanation for learning that occurs on behavioral time scales.

## Data Availability Statement

The datasets presented in this study can be found in online repositories. The names of the repository/repositories and accession number(s) can be found below: http://modeldb.yale.edu/266868 ModelDB Accession number 266868.

## Author Contributions

IC carried out simulations and analysis and wrote the initial drafts of the paper. HS conceived of the project and the analytical methods and edited the drafts. All authors contributed to the article and approved the submitted version.

## Conflict of Interest

The authors declare that the research was conducted in the absence of any commercial or financial relationships that could be construed as a potential conflict of interest.
