# Supplementary material for: Behavioral Time Scale Plasticity of Place Fields: Mathematical Analysis
Source: Front Comput Neurosci. 2021 Mar 1;15:640235. doi: 10.3389/fncom.2021.640235 (PMC7959845; doi:10.3389/fncom.2021.640235)
Supplement: Supplementary file 1 [file Data_Sheet_1.pdf]

APPENDIX

Analytical solution for rectangular place fields

In this section we drop for simplicity the superscript for type of trace and the subscript for synapse index. Using the trace equation (Equation 3) and the above choice of R, the integration factor U (see General trace solution) has the form:

$$U(t) = \begin{cases} e^{t/\tau} & : \quad t \leq t_1 \\ e^{\frac{1}{\tau}[(1+\eta\alpha)t - \eta\alpha t_1]} & : \quad t_1 < t < t_2 \\ e^{\frac{1}{\tau}[t+\eta\alpha(t_2-t_1)]} & : \quad t \geq t_2 \end{cases} \tag{A1}$$

Define $V(t) = \int_0^t R(t')U(t')dt'$. The solution (see Equation 12) with $C = 0$ is simply $T(t) = \eta T_{max} V(t)/(U(t)\tau)$. Let's now calculate $V(t)$. For $t \leq t_1$, $V(t) = 0$, because $R(t)$ is zero over this range. For $t_1 < t < t_2$:

$$V(t) = \frac{\eta\alpha\tau}{1+\alpha\eta} e^{-\eta\alpha t_1/\tau} \left[e^{(1+\eta\alpha)t/\tau} - e^{(1+\eta\alpha)t_1/\tau} \right]. \tag{A2}$$

For $t > t_2 : V(t) = V(t_2)$.

Therefore:

$$T(t) = \begin{cases} 0 & : \quad t \leq t_1 \\ \frac{T_{max}\eta\alpha}{1+\eta\alpha} \left[1 - e^{-\frac{1+\eta\alpha}{\tau}(t-t_1)} \right] & : \quad t_1 < t < t_2 \\ \frac{T_{max}\eta\alpha}{1+\eta\alpha} \left[1 - e^{-\frac{1+\eta\alpha}{\tau}(t_2-t_1)} \right] e^{-\frac{t-t_2}{\tau}} & : \quad t \geq t_2 \end{cases} \tag{A3}$$

Recall that one may take the standard trace equation (Equation 4) and transform it into the one with a basal level (Equation 3) via the changes of variables $T_i \rightarrow (T_i - T_0)$ and $T_{max} \rightarrow T_{max} - T_0$. So if we are to include a basal level in our analytical solution, all the T_{max} in Equation A3 become $T_{max} - T_0$, and the constant T_0 is added to the right side of the equation in all three cases.

Now that $T(t)$ has been solved, one may find the fixed point of W_i (Equation 10) by solving for I_i^k (Equation 8). Assume that we have an instructive signal of the form:

$$P(t - t_P) = \begin{cases} 0 : & t < t_P \\ \gamma e^{-\frac{t-t_P}{\tau_I}} : & t \geq t_P \end{cases} \tag{A4}$$

Where t_P is the time of the start of the instructive signal. Since $I_i^k = \int_0^{t_{trial}} T_i^k(t)P(t-t_P)dt$, $I_i^k = 0$ for both $t < t_1$ ($T_i^k(t) = 0$) and $t < t_P$ ($P(t - t_P) = 0$). This leaves us with three cases to integrate over: $t_P < t_1$, $t_1 \leq t_P < t_2$, and $t_2 \leq Tt_P < t_{trial}$.

For $t_P < t_1$:

$$I_i^k = \int_{t_1}^{t_2} \frac{\eta\alpha T_{max}}{1+\eta\alpha}\left[1-e^{-\frac{1+\eta\alpha}{\tau}(t-t_1)}\right]\gamma e^{-\frac{t-t_P}{\tau_I}}dt + \int_{t_2}^{t_{trial}} \frac{\eta\alpha T_{max}}{1+\eta\alpha}\left[1-e^{-\frac{1+\eta\alpha}{\tau}(t_2-t_1)}\right]e^{-\frac{t-t_2}{\tau}}\gamma e^{-\frac{t-t_P}{\tau_I}}dt \quad \text{(A5)}$$

which can alternatively be written as:

$$I_i^k = \frac{\gamma\eta\alpha T_{max}}{1+\eta\alpha}\left[\int_{t_1}^{t_2} e^{-\frac{t-t_P}{\tau_I}}dt - \int_{t_1}^{t_2} e^{-\left[\frac{1+\eta\alpha}{\tau}(t-t_1)+\frac{t-t_P}{\tau_I}\right]}dt + \int_{t_2}^{t_{trial}} e^{-\left[(\frac{1}{\tau}+\frac{1}{\tau_I})t-\frac{t_2}{\tau}-\frac{t_P}{\tau_I}\right]}dt - \int_{t_2}^{t_{trial}} e^{-\left[\frac{1+\eta\alpha}{\tau}(t_2-t_1)+\frac{t-t_2}{\tau}+\frac{t-t_P}{\tau_I}\right]}dt\right] \quad \text{(A6)}$$

The two other cases ($t_1 \le t_P < t_2$ and $t_2 \le t_P < t_{trial}$) can be solved via the equation above by substituting the limits of integration as appropriate. Following this prescription leads to the following solutions:

For $t_P < t_1$:

$$I_i^k = \frac{\gamma\eta\alpha T_{max}}{1+\eta\alpha}\left[\tau_I e^{t_P/\tau_I}\left(e^{-t_1/\tau_I}-e^{-t_2/\tau_I}\right) + \frac{e^{\frac{1+\eta\alpha}{\tau}t_1+t_P/\tau_I}}{\left(\frac{1+\eta\alpha}{\tau}+\frac{1}{\tau_I}\right)}\left[e^{-(\frac{1+\eta\alpha}{\tau}+\frac{1}{\tau_I})t_2}-e^{-(\frac{1+\eta\alpha}{\tau}+\frac{1}{\tau_I})t_1}\right] + \frac{e^{t_P/\tau_I+\frac{1+\eta\alpha}{\tau}t_1-\eta\alpha t_2/\tau}-e^{t_2/\tau+t_P/\tau_I}}{\left(\frac{1}{\tau}+\frac{1}{\tau_I}\right)}\left[e^{-(\frac{1}{\tau}+\frac{1}{\tau_I})t_{trial}}-e^{-(\frac{1}{\tau}+\frac{1}{\tau_I})t_2}\right]\right] \quad \text{(A7)}$$

For $t_1 \le t_P < t_2$:

$$I_i^k = \frac{\gamma\eta\alpha T_{max}}{1+\eta\alpha}\left[\tau_I\left(1-e^{-\frac{t_2-t_P}{\tau_I}}\right) + \frac{e^{\frac{1+\eta\alpha}{\tau}t_1+t_P/\tau_I}}{\left(\frac{1+\eta\alpha}{\tau}+\frac{1}{\tau_I}\right)}\left[e^{-(\frac{1+\eta\alpha}{\tau}+\frac{1}{\tau_I})t_2}-e^{-(\frac{1+\eta\alpha}{\tau}+\frac{1}{\tau_I})T}\right] + \frac{e^{t_P/\tau_I+\frac{1+\eta\alpha}{\tau}t_1-\eta\alpha t_2/\tau}-e^{t_2/\tau+t_P/\tau_I}}{\left(\frac{1}{\tau}+\frac{1}{\tau_I}\right)}\left[e^{-(\frac{1}{\tau}+\frac{1}{\tau_I})t_{trial}}-e^{-(\frac{1}{\tau}+\frac{1}{\tau_I})t_2}\right]\right] \quad \text{(A8)}$$

For $t_2 \leq t_P < t_{trial}$:

$$I_i^k = \frac{\gamma\eta\alpha T_{max}}{1+\eta\alpha}\left[\frac{e^{t_P/\tau_I+\frac{1+\eta\alpha}{\tau}t_1-\eta\alpha t_2/\tau}-e^{t_2/\tau+t_P/\tau_I}}{\left(\frac{1}{\tau}+\frac{1}{\tau_I}\right)}\left[e^{-(\frac{1}{\tau}+\frac{1}{\tau_I})t_{trial}}-e^{-(\frac{1}{\tau}+\frac{1}{\tau_I})t_P}\right]\right] \quad \text{(A9)}$$

For traces with a basal level, each of three cases has an additional basal overlap $I_0^k = \int_0^{t_{trial}} T_0^k P(t - t_P)dt = \gamma T_0^k \tau_I (1 - e^{\frac{t_P - t_trial}{\tau_I}})$.

The fixed point can then be calculated by plugging in these expressions for I_i^k into $W_i = \frac{I_i^p}{I_i^p + I_i^d}$ (Equation 10), for each of the three cases. Figure 9 shows the fixed point solution that results from these calculations, assuming a non-zero basal level for the LTD trace.
